# Dose Considerations for Vaccinia Oncolytic Virus Based on Retrospective Reanalysis of Early and Late Clinical Trials

**DOI:** 10.3390/vaccines12091010

**Published:** 2024-09-03

**Authors:** Mefotse Saha Cyrelle Ornella, Jae-Joon Kim, Euna Cho, Mong Cho, Tae-Ho Hwang

**Affiliations:** 1 Department of Pharmacology, School of Medicine, Pusan National University, Yangsan 50612, Republic of Korea; ornella@pusan.ac.kr; 2 Bionoxx Inc., Parkview Tower #1905, 248 Jeongjail-ro, Bundang-gu, Seongnam-si 13554, Republic of Korea; euna.cho@bionoxx.com (E.C.); mcho@bionoxx.com (M.C.); 3 Division of Hematology & Oncology, Department of Internal Medicine, Pusan National University Yangsan Hospital, Yangsan 50612, Republic of Korea; kimjj@pusan.ac.kr; 4 Medical Research Center, School of Medicine, Pusan National University, Yangsan 50612, Republic of Korea; 5 Department of Clinical Pharmacology, Pusan National University Yangsan Hospital, Yangsan 50612, Republic of Korea

**Keywords:** oncolytic viruses, vaccinia oncolytic virus, hormetic dose, OV-induced neutrophils, immune modulation

## Abstract

Over the past decade, oncolytic viruses (OVs) have been developed as a promising treatment alone or in combination in immuno-oncology but have faced challenges in late-stage clinical trials. Our retrospective reanalysis of vaccinia oncolytic virus (VOV) clinical trials indicates that lower doses—rather than the maximum tolerated dose (MTD)—are associated with better tumor response rates. Patients who responded well to lower doses generally had prolonged survival rates in the early phase clinical trial. The association between poor outcomes and an increase in OV-induced neutrophils (OV-N) but not baseline neutrophil counts suggests the need for a comprehensive characterization of OV-N. Although this reanalysis is limited by patient heterogeneity—including differences in cancer type and stage, treatment schedules, and administration routes—it remains informative given the complexities of translational studies in the tumor-bearing mouse models of vaccinia oncolytic viruses. Notably, while OV-N increases with higher viral doses, the immune state shaped by tumor progression likely amplifies this tendency. These findings highlight the importance of OV-N immune modulation as well as dose optimization for the successful clinical development of VOV.

## 1. Introduction

**Figure 1:**
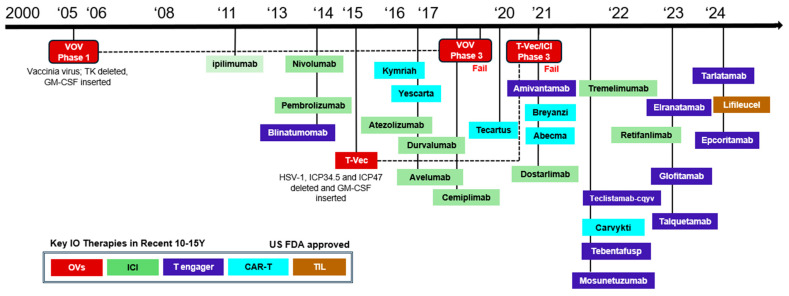
US FDA approval as a major milestone of immuno-oncology (IO) therapy. This graphic shows key IO therapy approvals from the US FDA. For OVs, noteworthy clinical trials have been included.

The field of immuno-oncology (IO) has shown significant advancements over the years with the introduction of US FDA-approved therapies such as immune checkpoint inhibitors (ICI) (e.g., anti-CTLA-4, anti-PD-1, and anti-PD-L1) [1], oncolytic viruses (OVs) like T-VEC (Talimogene laherparepvec) [2,3], chimeric antigen receptor (CAR)-T cell therapies [4], T cell engager therapies [5,6], and tumor-infiltrating lymphocytes (TIL) [7] (Figure 1). However, dose optimization primarily based on the traditional 3+3 dose-escalation design has been insufficient to determine the most appropriate dose due to a wide therapeutic window, an unclear dose–toxicity relationship, and different patient susceptibility [8]. In some cases, lower doses of ICI were required to be assessed during post market surveillance. In light of the need to select doses that prevent unnecessary toxicity and cost, the US FDA has also taken initiative and announced Project Optimus, a project to reform dose optimization and selection for oncology. The dose optimization for OVs also requires considerable insight because the MTD may or may not be determined, and more importantly, the individual host immune response makes it difficult to predict a clear dose–effect relationship similar to ICI.

Significant progress has been made in enhancing the specificity and efficacy of OVs through genetic engineering. However, the US FDA-approval of just one oncolytic virus and its recent failure when combined with ICI [9,10] highlights the need for more studies to understand the mechanisms of OVs in light of the host immune state or the immune response it provokes [11].

OVs are a promising new class of cancer biotherapeutics with antitumor effects conferred by diverse mechanisms of action. OVs selectively infect and replicate in tumor cells, inducing both lysis and host immune response [12,13]. OVs induce immunogenic cell death, increase the infiltration of tumor-specific CD8^+^ T cells, and render the tumor microenvironment (TME) more immunogenic [14,15]. The Vaccinia Virus (VACV) has been used as an attractive oncolytic virus backbone because of its well-established safety profile due to its long history of use as a smallpox vaccine, its ability to selectively target and infect tumor cells, and its potential to stimulate the immune system to attack cancer cells. Vaccinia oncolytic virus (VOV) has been administered to more than 400 patients through clinical studies since the 2000s, and its pharmacokinetics (PK), pharmacodynamics (PD), and immunological characteristics have been partially elucidated [16,17,18]. One of the most advanced VOVs in clinical development was engineered by removing the vaccinia virus thymidine kinase (VV-tk) from the Wyeth strain of VACV and replacing it with a human granulocyte–macrophage colony-stimulating factor (hGM-CSF) gene. Although some patients in the early clinical trials of the VOV showed promising clinical outcomes, including durable tumor response, subsequent phase 2b (NCT01387555) and phase 3 (NCT02562755) studies did not demonstrate clinical benefits in OS or tumor response [16,18,19,20]. Therefore, a reverse translational study for gap analysis between the early and late phases of VOV trials is necessary.

To understand the gap between early and later clinical trials, we conducted a retrospective reanalysis of the clinical trials of the TK-deleted and GM-CSF-inserted VOV, having used different names: vaccinia/GM-CSF recombinant virus [20], JX-594 [16,17], and Pexa-Vec (Pexastimogene Devacirepvec) [18,19,21,22], all of which were derived from the same master viral bank. One key component that may have contributed to the unsuccessful outcome of later-stage trials of VOVs is the dosing strategy employed and a gap in the mechanistic understanding of VOVs. The traditional 3+3 dose-escalation design that determines the maximum tolerated dose (MTD) is frequently employed by anticancer medications (chemotherapies) to optimize antitumor effects within the limitations of tolerability. However, oncolytic virotherapy may follow a different paradigm. We hypothesize that a non-monotonic dose–response curve, also called hormesis—a phenomenon that describes a dose–response curve where low doses enhance therapeutic benefit effects, while high doses reduce the effectiveness or even cause harm [23,24]—could potentially be applied to oncolytic virus (OV) treatments like VOV. This implies that the optimal therapeutic dose of VOV may be lower than the MTD, which would enable the harmonization of viral replication, the absence of virus-induced suppressive innate immune cells, tumor cell destruction, and immune activation to achieve greater therapeutic efficacy.

In this paper, we conducted a retrospective reanalysis of existing data on the PK and PD following VOV therapy, and suggest the importance of OV-induced neutrophils (OV-N) immune modulation and optimizing the viral dose by applying the hormetic dose–response principle.

## 2. Overview of PK and PD Following VOV Intratumoral Injection

Since the 2000s, clinical investigations have delivered VOV to over 400 patients, elucidating its PK, PD, and partly immunological properties [16,17,18]. The phase 1 study of VOV of the Wyeth strain, with granulocyte–macrophage colony-stimulating factor (GM-CSF) and beta-galactosidase transgene in place of the VV-tk gene (NCT00629759), provided important insights into the pharmacokinetics (PK) and pharmacodynamics (PD) of VOV. Following the intratumoral injection of VOV, released virus particles were detected in the blood shortly after injection and decreased by 50% within 15 min and more than 90% within 4–6 h [16]. The quantity of VOV in blood showed a dose-dependent pattern, indicating that a constant proportion was released systemically during the direct injection. Between days 3 and 22 post-injection, a delayed re-emergence of circulating VOV was observed in some patients, which may be unrelated to the administered doses. Although neutralizing antibody (nAb) production was not consistent across all patients, a maximum nAb level is generally reached at 3 weeks post injection [16]. Even with repeated administration, a secondary peak of virus replication was observed. Partial response and complete response were achieved in patients having received multiple injections (>6 cycles). This indicates that nAb production does not have a significant impact on the biopharmaceutical properties of OVs’ intratumoral injection, and therefore, repeated administration after neutralizing antibody production is still viable.

VOV replicates selectively in tumor cells but can also redistribute into the bloodstream as well as to non-injected masses after intratumoral injection [16]. As replication-competent VOV does not follow the typical absorption, distribution, metabolism, and excretion (ADME) process, immunological factors such as activated immune cells may play a more important role in the distribution of VOV than the administered viral dose (NCT00629759) [16]. The mechanisms of action of VOV include the direct oncolysis by cancer specific replication [16,17,25], vascular disruption [26], and more importantly, the production of antitumor immune response for substantial tumor response and long-term survival [18,27]. Promising clinical results have shown that VOV treatment increases tumor necrosis factor (TNF-α) levels and IFN-γ at tumor sites. This elevation leads to the activation and recruitment of neutrophils, eosinophils, and lymphocytes to the tumor tissues, suggesting immune activation [18,21,27,28]. However, depending on the patients’ immune status, the sustained, excessive production of ANC (absolute neutrophil count) after VOV administration, defined here as oncolytic virus-induced neutrophils (OV-N), in the peripheral blood may be relevant to severe adverse events as well as limited tumor response.

The reason we speculate that the patient’s immune state influences the OV-N response is that, while higher doses generally lead to a greater increase in ANC (absolute neutrophil count), this is not always consistent. Additionally, animal studies have shown that the pattern of activated neutrophils following VOV administration differ between tumor-bearing mice and non-tumor-bearing mice (unpublished data).

## 3. Retrospective Reanalysis of VOV Clinical Outcome

Several clinical trials have been conducted to assess the safety and efficacy of VOV, and the clinical study designs of VOV used in the retrospective reanalysis are listed in the following Table 1. This review included all published clinical trials performed on the TK-deleted and GM-CSF-inserted VOV, regardless of the treatment schedule and disease stage, where the therapy was solely VOV monotherapy. Also, out of all the trials, most of them involved the use of intratumoral injections of VOV, although in the TRAVERSE study (NCT01387555), patients were given one intravenous injection at first. Early clinical trials of VOV showed promising outcomes, including durable tumor response; however, phase 2b and phase 3 studies revealed no clinical benefit in terms of overall survival (OS) or tumor response [16,18,19,20,29]. Consequently, a reanalysis of these clinical trials was conducted [16]. The dose-escalation phase 1 trial showed that VOV was well tolerated in patients with advanced liver cancer (both primary and metastatic liver cancer). VOV was administered at a dose ranging from 1 × 10^8^ to 3 × 10^9^ pfu [16,17,19,21,22]. The maximum tolerated dose (MTD) was determined to be 1 × 10^9^ pfu when direct administration into the tumor resulted in dose-limiting toxicity (DLT) at 3 × 10^9^ pfu. In a randomized phase 2 clinical trial of VOV, a dose of 1 × 10^9^ pfu demonstrated a clinical benefit in increasing the survival compared to a dose of 1 × 10^8^ pfu in 30 patients with primary liver cancer who had not been exposed to sorafenib [18]. However, in a phase 2b clinical trial (NCT01387555) involving patients with sorafenib-resistant liver cancer, despite a tolerable safety profile and induction of T cell responses, VOV did not improve the OS as a second-line therapy after sorafenib failure [19]. Similarly, a phase 3 pivotal study (NCT02562755) comparing VOV plus sorafenib versus sorafenib alone in hepatocellular carcinoma did not demonstrate improved overall survival.

**Figure 2:**
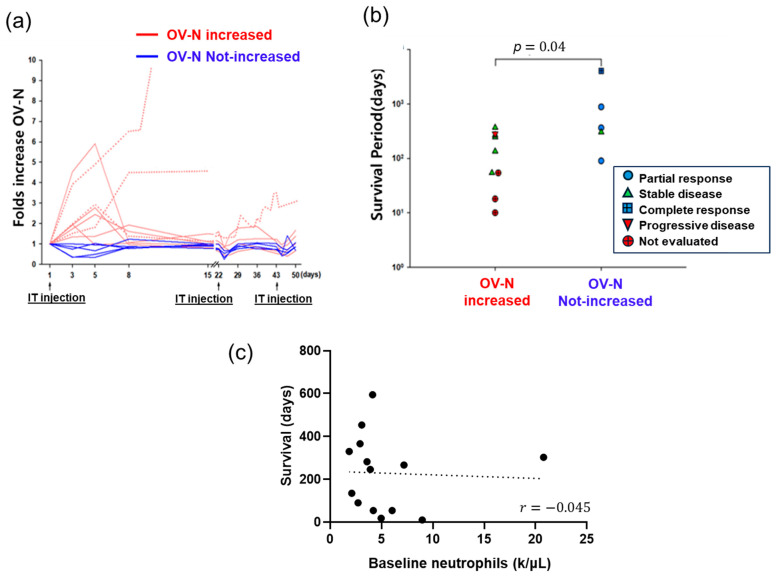
Temporal profile of neutrophil changes and their relationship with tumor response: reanalysis of phase 1 study of VOV in patients with refractory liver cancer (NCT 00629759). Increased neutrophil counts after VOV therapy are associated with decreased survival, particularly in patients treated with higher doses (1 × 10⁹) of VOV. (**a**) Temporal profile of ANC changes. (**b**) Relationship of temporal profile of ANC changes with tumor response and survival benefit. (**c**) Correlation between baseline neutrophil and survival.

Our reanalysis of early clinical trials conducted with the intratumoral injection of a single agent revealed that increasing the dosage of VOV did not enhance its efficacy, contrary to the conventional dose–response relationship observed in cancer chemotherapy. Instead, higher doses resulted in a higher percentage of severe systemic toxicity and decreased efficacy. In various solid tumor patients, a greater proportion of patients receiving lower doses (10 out of 26 patients) had a durable tumor response compared to those receiving the MTD or higher doses (1 out of 88 patients) (Table 2). Furthermore, in the phase 1 dose-escalation study of VOV (NCT00629759), nine patients experienced an increase in the absolute neutrophil count (ANC), and these ANC increases were negatively related to the tumor response and survival time (Figure 2). In addition, good tumor responses were observed in patients with a transient decrease or no change in ANC *(n* = 5). Although the number of patients was limited (*n* = 14) and four different doses were used, clinical benefit was negatively related with sustained ANC increase. Furthermore, sustained high levels of OV-N could contribute to systemic inflammation, which could have a negative impact on clinical outcomes and be correlated with adverse effects. However, no other immunological factors, biochemistry tests, or even baseline ANC were correlated with clinical outcomes (Table 2). This reanalysis demonstrates the significance of determining the appropriate therapeutic dose in order to achieve a balance between “efficacy and toxicity” and the importance of controlling OV-N.

## 4. Proposed Hormetic Dose–Response

Almost all substances are toxic when administered in sufficiently large quantities [30]. The term “hormesis”, derived from the Greek word “hormáein”, is a phenomenon where small doses of therapeutic agents, such as drugs, immune reactants, or radiation, stimulate beneficial effects, while higher doses lead to inhibitory or harmful effects. This dual nature of hormesis poses significant challenges for making treatment decisions in cancer therapy [31,32,33,34]. Nevertheless, hormesis has been investigated in many cancer treatment studies, where hormetic effects, alone or in combination with synergistic effects, have led to improved tumor response and reduced treatment toxicity [35].

**Figure 3:**
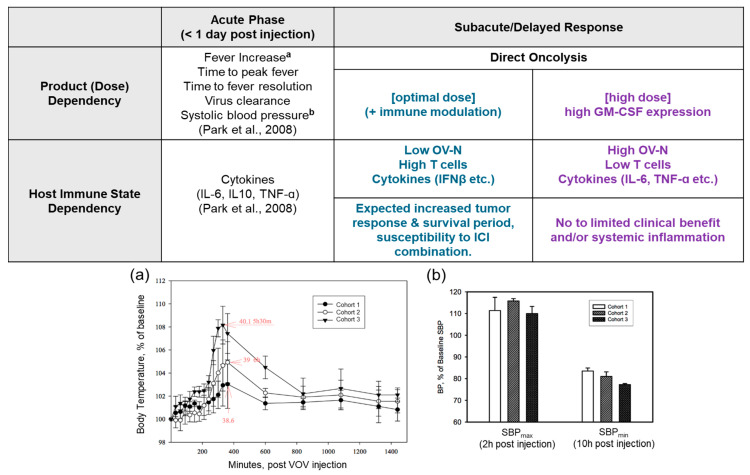
Proposed scheme of dose or host immune state-dependent acute and subacute response post-VOV treatment. The optimal dose (blue) of VOV, in contrast to the high dose (purple), may harness a favorable host-dependent subacute responses, especially in combination with immunomodulatory drugs. (**a**) Dose-dependent level of maximum body temperature. (**b**) Systolic blood pressure profiles <1 day post VOV administration. SBP: systolic blood pressure [16].

Based on the analysis of clinical parameters, we observed that, after VOV administration, the host response can be divided into acute (<1 day post viral injection) and subacute/delayed responses (Figure 3). In the phase 1 study (NCT00629759), acute responses that occur in a dose-dependent manner, such as viral particle count and time-to-peak fever, may not be directly correlated with clinical outcomes. The proposed hypothesis (Figure 3) is that subacute responses, which involve an increase in OV-N, and reduced T cell activation, may directly affect clinical outcomes. The objective of immune regulation by means of immunomodulatory drugs may be associated with the subacute host response to VOV treatment. Using immunomodulatory drug therapy in combination with VOV may minimize the OV-N adverse effect and maximize anticancer effects.

Specifically, while there was no tumor response (mRECIST) in the phase 2b (TRAVERSE) study of the maximum tolerated dose (MTD) of VOV (i.t., 1 × 10^9^ pfu/dose) against refractory liver cancer, durable tumor response and survival were observed in phase 1 and phase 2a with doses below the MTD. Among various factors analyzed including complete blood count (CBC) parameters, serum biochemistry, cytokine changes, adaptive immune cell changes, fever patterns, secondary virus replication, and neutralizing antibody production, only an increase in neutrophils after VOV administration was correlated with poor clinical outcomes. Baseline neutrophil count was not associated with survival benefit (Figure 2). They are interesting because they may reflect the recently highlighted functional heterogeneity of circulating neutrophils increased after the administration of immunotherapy [36].

**Figure 4:**
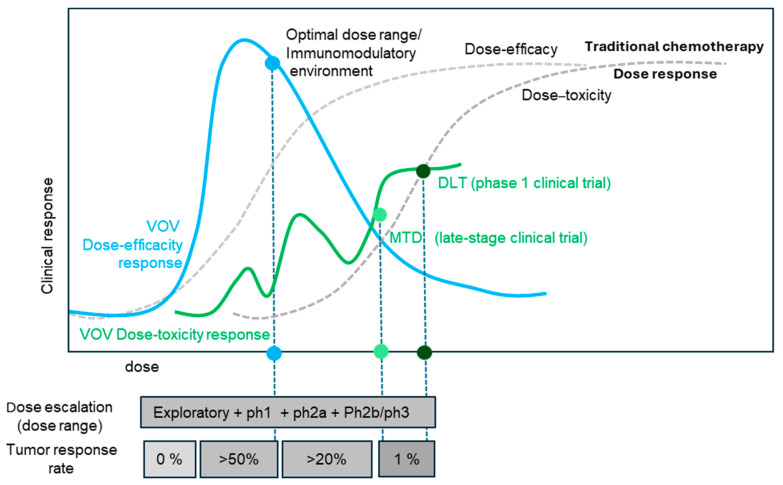
Proposed dose–response curve of VOV based on a retrospective reanalysis of clinical trials. The potential hormetic dose–response relationship of VOV is illustrated in comparison to that of traditional chemotherapies. The fluctuating nature of the VOV dose–toxicity response curve is influenced by host factors such as the immune system’s response to the virus, which can contribute to toxicity independently of the dose itself. This illustration is supported by the literature on hormesis and non-monotonic dose–response relationships in cancer therapy, as well as this retrospective analysis of VOV.

We hypothesize that, unlike chemotherapy, the efficacy of VOV treatment relies on a non-monotonic dose–response. Therefore, optimizing the VOV dose at a lower level, instead of the MTD, may be more effective and also reduce the risk of severe treatment-related toxicity (Figure 4). This theory is supported by the literature on hormesis and non-monotonic dose–response relationships in cancer therapy [23,31,32,33] as well as this retrospective analysis of VOV. Furthermore, decreased efficacy at higher doses of VOV might be attributed to systemic inflammation via sustained OV-N, which can suppress the OVs replication and adaptive immunity. Moreover, the suppression of neutrophils may increase activated tumor-specific T cell immunity [36,37,38,39,40], leading to enhanced antitumor efficacy and a wider therapeutic window (Figure 3).

## 5. Discussion

Optimizing the dose of immuno-oncology therapies as well as implementing strategies for host immune modulation is crucial due to the complexities of the mechanisms underlying their therapeutic effects [11]. Dose optimization strategies for ICIs and CAR-T cells are still being debated because the lack of MTD, individual immune response, and contradictions related to the dose–toxicity or dose–efficacy response [41]. The dose–efficacy of each treatment may be influenced by factors like the tumor microenvironment, immune state, and individual patient heterogeneity, which suggest that the dose selection may need to consider these factors. An alternate model for the dose–response relationship, corresponding to the underlying mechanisms of action, is therefore essential for enhancing clinical benefit.

In the context of vaccinia oncolytic virus (VOV) clinical trials, the heterogeneity of patients reflected in differences in cancer type, disease stage, treatment schedules, and administration routes (including intravenous injection in NCT01387555 trial) presents certain limitations when making cross-trial comparisons. Although a more widely researched association between VOV dose and clinical response is warranted, this retrospective reanalysis offers a unique perspective of VOV clinical trial data where the VOV was assessed as a single agent and was mainly administered intratumorally. Additionally, this may be particularly valuable due to the challenges of translating preclinical findings to human patients, especially in terms of the differences in virus permissiveness, immune response to VOV treatment, and the tumor/systemic immune environment.

Despite the variability, our retrospective reanalysis illustrates that patients who responded well showed prolonged survival, particularly in early clinical stages in which patients received a lower dose of VOV. In contrast, late-stage trials did not demonstrate significant therapeutic benefit in terms of overall survival or tumor response. Such findings suggest that lower doses of VOV may be more effective than higher doses or the maximum tolerated dose (MTD), potentially indicating a hormetic dose–response relationship. While PK and onset of fever were strictly correlated with virus dose, they were not correlated with a clinical response. Therefore, the host immune factor may be more decisive in subsequent durable tumor response. This observation raises further questions about the role of OV-induced neutrophils (OV-N) in treatment outcomes. Specifically, our analysis showed a correlation between the poor clinical outcome and increased OV-N levels, but a correlation was not observed with baseline neutrophils. The relationship between the VOV dosage and OV-N increase appear to be complex and may not be solely attributable to the dose; the production of GM-CSF by VOV and its potential link to the OV-N increase remains unclear [16]. Notably, clinical trials involving the Western Reserve vaccinia virus (which does not encode GM-CSF) also reported increased absolute neutrophil counts (ANCs) in some cases, potentially due to TLR-mediated neutrophil activation [42].

Further investigation is warranted to fully realize the potential of VOV and other OVs as monotherapy or combination. The comprehensive characterization of OV-N to better understand their impact on the host response and the development of physiological immune modulations may be key in enhancing treatment efficacy. Furthermore, several early phase clinical studies are underway to investigate the safety and efficacy of the combination treatment of VOV and immune checkpoint inhibitors (ICIs). Combination of VOV with durvalumab and tremelimumab has shown a safe, tolerable effect and potential antitumor activity in patients with a proficient mismatch repair metastatic colorectal cancer (NCT03206073) [43]. VOV with cemiplimab has also shown an acceptable safety profile and efficacy in patients with metastatic or unresectable renal cell carcinoma (NCT03294083) [44]. VOV in combination with an anti-PD-L1 monoclonal antibody (ZKAB001) for metastatic melanoma (NCT04849260) is currently recruiting. The clinical trial of VOV with ipilimumab for metastatic/advanced solid tumors (NCT02977156) is now complete and the results are pending. These efforts may be more fruitful with an understanding of the criticality of OV-N and the optimization of immune modulation upon combination.

Finally, the recent acceptance by the US FDA of Bionoxx’s Investigational New Drug (IND) application to initiate the first-in-human trial of mOTS-412—an engineered oncolytic vaccinia virus [45] utilizing immune modulation to suppress OV-N—underscores the development path for this therapeutic strategy. This trial will evaluate the safety and efficacy of mOTS-412 and in combination with anti-PD-L1 in patients with tumors resistant to ICIs. This development highlights the potential for advancing immune modulation strategies targeting resistant tumors, an area that warrants continued research to improve patient outcomes. Alternative administration routes such as intravenous injection also merit exploration once the systemic immune state has been elucidated via in-depth translation studies. The insights gained from VOV studies may also inform the optimization of other OVs, guiding cross-viral research to improve the treatment outcomes across different oncolytic platforms.

## 6. Conclusions

Our retrospective reanalysis of previous VOV clinical trials revealed that lower doses of VOV might be more optimal than higher doses or the maximum-tolerated dose (MTD), suggesting a hormetic dose–response relationship. Additionally, we found a correlation between poor clinical outcomes and an increase in OV-induced neutrophils (OV-N), but not baseline neutrophil counts. This suggests that the dose adjustment (to lower doses) of VOV may allow for the optimization of viral replication, tumor lysis, and immune activation while avoiding the immunosuppressive effects associated with higher doses. These demonstrate the significant need to reconsider the dose strategies a nd conduct further research on the characterization and regulation of OV-N to enhance the outcomes of oncolytic virotherapy for cancer patients.

## Figures and Tables

**Table 1:** Study design of POC, phase 1, 2a, 2b clinical studies of intratumoral VOV utilized in the retrospective reanalysis.

Phase	Disease	No. of Pts *	Dose/Cycle	Primary Objective	Conclusion	Ref.
Proof of concept	Melanoma	**7**	1 × 10^4^~8 × 10^7^ pfu (i.t.)/twice weekly, for 6 weeks	Evaluation of safety and efficacy of Pexa-Vec	Injected and non-injected tumor lesions regressed Toxicity was minimal **Tumor response: 71.4%**	[20]
1 Dose-escalation (NCT00629759)	Primary/metastatic liver cancer	**14**	1 × 10^8^, 3 × 10^8^, 1 × 10^9^, 3 × 10^9^ pfu (i.t.)/4 cycles (every 3 weeks)	Evaluation of safety of Pexa-Vec Determine MTD and/or MFD	MTD of 10^9^ pfu was confirmed; mild-to moderate flu-like symptoms appeared as the most common adverse event **Tumor response: 21.4%**	[16]
2a Randomized dose-finding (NCT00554372)	HCC	**28**	1 × 10^8^, 1 × 10^9^ pfu (i.t.)/3 cycles (every 2 weeks)	Proportion of subjects achieving disease control (non-progressive disease) at 8 weeks after initiation of treatment	Survival duration was significantly dose-related, in contrast to tumor response and immune endpoints **Tumor response: 14.3%**	[18]
2b (TRAVERSE) (NCT01387555)	HCC	**65**	1 × 10^9^ pfu (i.v.)/day 1 f/b 1 × 10^9^ pfu (i.t.)/5 cycles (day 8, week 3, 6, 12, and 18)	Overall survival (OS)	Pexa-Vec did not improve OS as second-line therapy after sorafenib failure **Tumor response: 0.0%**	[19]

* The number of patients indicates those treated at least once and that were evaluable. MTD: maximum tolerated dose; MFD: maximum feasible dose; i.v.: intravenous; i.t.: intratumoral; f/b: followed by; HCC: hepatocellular carcinoma.

**Table 2:** Retrospective investigators’ reanalysis of clinical outcome according to dosage of VOV IT injection.

Dose ^a^	Tumor Response (RECIST) ^b^ %(*n*)	Disease	Original Description on Systemic Toxicity	Investigators’ Retrospective Reanalysis	Ref.
1 × 10^4^–8 × 10^7^	71.4% (5/7 pts)	Melanoma	Flu-like symptom with ≥4 × 10^7^ pfu, 12% No hematological and ANC level change	N/A	[20]
1 × 10^8^–3 × 10^8^	26.3% (5/19 pts)	Lung/thymic, colon, gastric, liver, extragonadal	Mild flu-like symptoms (grade 1–2) No grade 4–5 Adverse effect	Tumor response is associated with ANC decrease post treatment Adaptive tumor immunity was confirmed in long-time survivors with complete response	[16,18,27]
1 × 10^9^–3 × 10^9^	1.1% (1/88 pts)	Renal, colon, liver, melanoma	Treatment-related DLT - Grade 3 anorexia and abdominal pain - Grade 3 hyperbilirubinemia	DLT may be associated with severe systemic inflammation following sustained ANC increase post treatment	[16,18,19]

^a^ Patients from the same dose range were combined from studies NCT00629759, NCT00554372, and NCT01387555. ^b^ includes complete, partial, mixed-response. pfu: plaque-forming unit; DLT: dose-limiting toxicity; ANC: absolute neutrophil count; N/A: not applicable.
